# Excess mortality in U.S. prisons during the COVID-19 pandemic

**DOI:** 10.1126/sciadv.adj8104

**Published:** 2023-12-01

**Authors:** Naomi F. Sugie, Kristin Turney, Keramet Reiter, Rebecca Tublitz, Daniela Kaiser, Rebecca Goodsell, Erin Secrist, Ankita Patil, Monik Jiménez

**Affiliations:** ^1^ Department of Criminology, Law and Society, University of California, Irvine, Irvine, CA 92697, USA.; ^2^ Department of Sociology, University of California, Irvine, Irvine, CA 92697, USA.; ^3^ Institute for State and Local Governance, City University of New York, New York, NY 10016, USA.; ^4^ Brigham and Women’s Hospital, 75 Francis Street, Boston, MA 02115, USA.

## Abstract

U.S. prisons were especially susceptible to COVID-19 infection and death; however, data limitations have precluded a national accounting of prison mortality (including but not limited to COVID-19 mortality) during the pandemic. Our analysis of mortality data collected from public records requests (supplemented with publicly available data) from 48 Departments of Corrections provides the most comprehensive understanding to date of in-custody mortality during 2020. We find that total mortality increased by 77% in 2020 relative to 2019, corresponding to 3.4 times the mortality increase in the general population, and that mortality in prisons increased across all age groups (49 and under, 50 to 64, and 65 and older). COVID-19 was the primary driver for increases in mortality due to natural causes; some states also experienced substantial increases due to unnatural causes. These findings provide critical information about the pandemic’s toll on some of the country’s most vulnerable individuals while underscoring the need for data transparency and standardized reporting in carceral settings.

## Teaser

U.S. prison mortality increased 77%, 3.4 times the general population mortality increase, in 2020, the year of COVID-19 onset.

## INTRODUCTION

Coronavirus disease 2019 (COVID-19) has differentially and disproportionately affected populations who were most vulnerable before the pandemic. While mortality in the United States increased by 23% between March 2020 and January 2021 (*1*), individuals with health risks, the economically disadvantaged, and racial/ethnic minorities have been disproportionately harmed by COVID-19 (*2*). Prisons, along with other “closed” institutions (e.g., nursing homes, immigration detention facilities, and jails) that concentrate these vulnerabilities into a single facility, emerged as epicenters of COVID-19 infection and death (*3*, *4*). U.S. prisons were sites of 39 of the country’s 50 largest COVID-19 outbreaks in 2020 (*5*), and COVID-19–related deaths are higher among people in prison than among those in the general population (*6*, *7*).

Still, the extent of total mortality in U.S. prisons during the pandemic is not known. Data from individual states provide some evidence of high mortality in prisons during the pandemic. For example, data from the Florida Department of Corrections show that all-cause mortality in prisons increased by 40% in 2020 (compared to 2019) and that life expectancy declined by 4 years (*8*). However, findings from individual states are not easily generalizable to the entire United States since state prison systems responded with different COVID-19 mitigation approaches (*9*, *10*). For COVID-19 deaths, specifically, the Bureau of Justice Statistics (BJS) recently reported 2490 deaths attributable to COVID-19 (suspected or confirmed as a cause of death) from March 2020 to February 2021 across 49 states and the Federal Bureau of Prisons (BOP), corresponding to 1.5 deaths per 1000 prisoners (*11*). But evidence suggests that focusing on COVID-19 deaths in prisons undercounts the total mortality toll of the pandemic. COVID-19 deaths are both underreported and fail to capture the likely increases in rates of collateral, non–COVID-19 deaths during the pandemic (*12*–*14*). Despite the Death In Custody Reporting Act, there is currently (2019 to present) no systematic reporting of total deaths (including deaths from other natural and unnatural causes) in U.S. prisons.

Not only are incarcerated people more vulnerable to COVID-19 (*3*, *4*), but COVID-19 policies in prisons may have exacerbated risk of death from other causes (*2*, *15*, *16*). Therefore, all-cause mortality rates among incarcerated people likely increased during the pandemic, as a result of both COVID-19 infection and pandemic-related changes that transformed the confinement experiences of incarcerated people. First, prisons have dense living arrangements that make social distancing difficult, limit resources for personal protective equipment and other infection-mitigation strategies, and generate frequent movement of staff between facilities and communities, exacerbating the risk of COVID-19 infection and death (*17*–*19*). Second, prisons faced unprecedented constraints on their staff and their medical resources, limiting access to routine primary and specialty medical care and delaying the timely delivery of care (*20*), exacerbating the risk of deaths due to natural causes, including but not limited to COVID-19. Third, prisons imposed policies attempting to mitigate infection—such as lockdowns and restricted movements, programming suspensions, visitor prohibitions, limited communication with loved ones, and solitary confinement in lieu of medical isolation—all of which increased stress, mental health challenges, and violence (*2*, *15*, *16*, *20*, *21*, *22*, *23*), exacerbating the risk of deaths due to unnatural causes, such as drug overdoses, suicide, and violence.

Here, we estimate excess mortality in U.S. prisons during the pandemic using data collected via systematic public records requests, a process involving multiple rounds of requests, responses, and follow-ups with Departments of Corrections (DOCs) across the United States. We supplement these data with publicly available data—including DOC statistical reports, public records requests made by other investigators, and third-party data sources—when necessary due to state nonresponses or refusals to provide requested data (see table S1 for a list of data sources and years observed for each DOC). These data are different from those reported by BJS (*11*), because they identify all deaths during the pandemic year of 2020, as opposed to deaths attributed to COVID-19 specifically. In total, we received mortality data from 49 DOCs (including 47 states, the Federal BOP, and Washington, D.C.); however, some states did not provide custody information or more detailed mortality information (by death month, age group, and/or manner of death). To be as comprehensive as possible, each analysis includes any state with relevant data; the resulting analytic sample varies depending on analysis and data availability (and we note which states are included in each analysis). The primary analytic sample, which provided information on annual counts of prison mortality and custody population throughout 2013–2020, includes 48 DOCs (including 46 states, the Federal BOP, and Washington, D.C., and excluding Vermont, which provided mortality but not custody population information). We also analyze different subsets of states to explore state characteristics associated with state-level variation in prison mortality rates, state-level variation by age, and excess mortality by manner of death. This variability in analytic samples highlights the very inconsistencies in prison death data reporting our public information requests and analysis document and address.

We focus on three primary research aims. First, we estimate excess total mortality for 2020 compared to 2019 and prior years. We present total mortality rate ratios (RRs) overall and by state, and we estimate negative binomial regression models, predicting deaths based on year fixed effects and exposure (custody) with clustered standard errors for states. In additional regression models with a subset of states (*N* = 46 with yearly data; *N* = 18 with monthly data), we include pre-pandemic (imprisonment rates and health care index) and pandemic-related (general population COVID-19 positivity rates) state-level variables to estimate their associations with total mortality. Although these variables capture only a portion of the possible factors influencing mortality in prisons, this additional analysis examines some key mechanisms potentially underlying increased deaths in custody. Additionally, in a subset of states with total mortality and custody information by age groups (*N* = 11), we estimate age-stratified RRs. Second, we estimate excess mortality by manner of death for 2020 compared to prior years. We follow a similar approach as above but distinguish between deaths due to natural and unnatural causes. Because of the recency of the pandemic, some states (*N* = 15) have large numbers of “unknown” deaths (10% or more of their total deaths in 2020). In additional analyses, we estimate mortality by manner of death without those states. Third, we estimate excess mortality for deaths due to natural causes other than COVID-19–related reasons. Again, we estimate mortality due to natural causes other than COVID-19 without states with large numbers of unknown deaths in 2020.

## RESULTS

Across 49 DOCs (including 47 states, the Federal BOP, and Washington, D.C.), the number of people who died in U.S. prisons was substantially higher in 2020 (6088 deaths) than in 2019 (4206 deaths) (see table S2). This comparison of total deaths does not account for changing custody populations, which decreased during the 2020 pandemic; therefore, rates of in-custody deaths were even higher in 2020. For DOCs with information about manner of death in 2020 (*N* = 41 DOCs, *N* = 5134 deaths), 4118 deaths (80%) were due to natural causes, 534 deaths (10%) were due to unnatural causes, and 482 deaths (9%) were due to unknown causes. For DOCs with information about COVID-19–related deaths (*N* = 19 states, *N* = 1714 total deaths), 496 deaths (29%) were related to COVID-19.

### Total mortality in 2020 compared to 2019

**Fig. 1.**
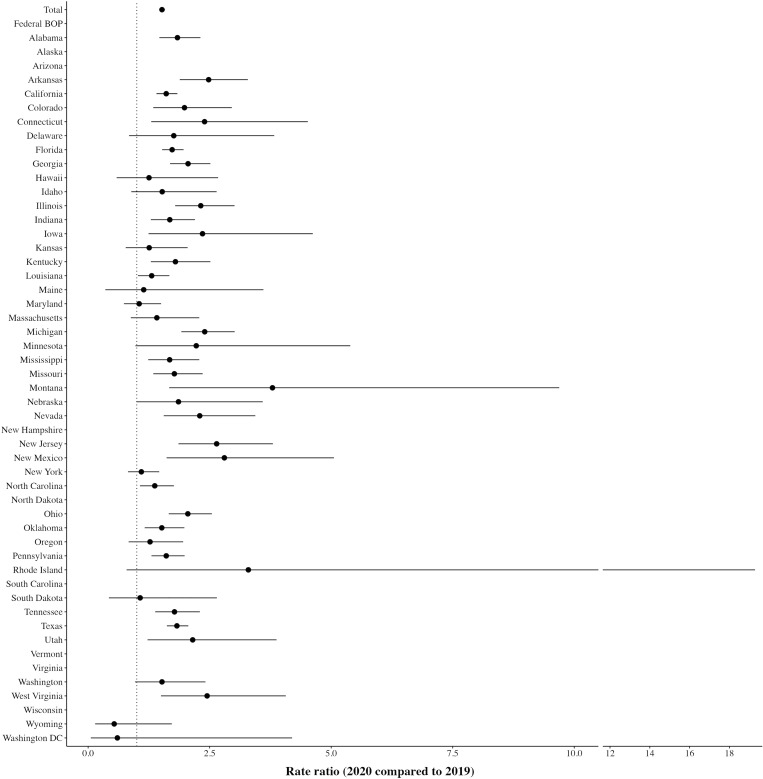
Total mortality rate ratio (2020 compared to 2019).

First, we estimate how the all-cause mortality rate changed between 2020 and 2019. We calculate the total mortality RR, overall and by state (Fig. 1). Across all states, the mortality rate in 2020 was 52% higher than 2019 [95% confidence interval (CI), 1.46 to 1.58]. This rate, however, masks substantial variation across states. In some states and jurisdictions (Maryland, New York, South Dakota, Wyoming, and Washington, D.C.), the RR is approximately equal to 1 or less. In other states, the total mortality RR is much higher, with many states (Arkansas, Connecticut, Georgia, Illinois, Iowa, Michigan, Montana, Nevada, New Jersey, New Mexico, Ohio, Utah, and West Virginia) experiencing a doubling, or more, of their mortality rate in 2020 compared to 2019. Table 1 (model 1) presents the results of a negative binomial regression analysis for total deaths with year fixed effects (2013–2019, with 2019 as a reference year) and clustered standard errors for 49 DOCs (47 states, including Arizona as an additional state for 2013–2019 and excluding Vermont, the Federal BOP, and Washington, D.C.). In these models, which account for clustering by state, the mortality RR for 2020 compared to the reference year of 2019 is 1.77 (CI: 1.62 to 1.93, *P* < 0.001).

**Table 1.** Negative binomial regression model estimating mortality, total and by manner. Estimates are based on negative binomial models, with clustered standard errors by state. End of year custody counts are imputed for Alaska, BOP, New Hampshire, North Dakota, and South Carolina.

	Model 1: Total mortality				Model 2: Natural causes				Model 3: Unnatural causes			
	Coefficient	RR	95% CI	*P*	Coefficient	RR	95% CI	*P*	Coefficient	RR	95% CI	*P*
Year (reference = 2019)												
2020	0.57	1.77	(1.62–1.93)	<0.001	0.59	1.80	(1.59–2.05)	<0.001	0.08	1.08	(0.93–1.27)	0.308
2018	0.00	1.00	(0.94–1.07)	0.916	0.03	1.03	(0.95–1.13)	0.444	−0.13	0.87	(0.75–1.01)	0.076
2017	−0.02	0.98	(0.91–1.05)	0.550	0.03	1.03	(0.94–1.11)	0.568	−0.10	0.91	(0.74–1.12)	0.370
2016	−0.02	0.98	(0.91–1.06)	0.568	0.05	1.05	(0.95–1.16)	0.306	−0.28	0.76	(0.62–0.93)	<0.01
2015	−0.06	0.94	(0.87–1.02)	0.129	0.06	1.06	(0.98–1.14)	0.135	−0.60	0.55	(0.46–0.66)	<0.001
2014	−0.19	0.82	(0.74–0.91)	<0.001	−0.14	0.87	(0.76–1.00)	<0.05	−0.54	0.59	(0.45–0.76)	<0.001
2013	−0.21	0.81	(0.73–0.89)	<0.001	−0.13	0.88	(0.76–1.01)	0.066	−0.84	0.43	(0.34–0.55)	<0.001
Constant	−5.70	0.00	(0.00–0.00)	<0.001	−5.97	0.00	(0.00–0.00)	<0.001	−7.47	0.00	(0.00–0.00)	<0.001
DOCs (N)			49			41				41		

Additionally, to examine mechanisms underlying increased deaths in custody, we estimate regression models with a subset of states (*N* = 46 in yearly models, *N* = 18 in monthly models) and a subset of years (2019, 2020) that include state-level measures of imprisonment rates, a prison health care index, and general population COVID-19 positivity rates. The findings (table S3) indicate that the state imprisonment rate is positively associated with mortality overall but is not related to mortality increases in 2020 (in yearly models). The state prison health care index is not associated with mortality. Notably, general population COVID-19 positivity rates are associated with total mortality (RR = 1.03, CI: 1.01 to 1.05, *P* < 0.01) in monthly models, indicating that a state’s monthly COVID-19 positivity rate is related to increases in the state DOC’s mortality rate in the following month.

We also calculated age-stratified total mortality RRs for three age groups: 49 years and under, 50 to 64 years, and 65 years and older. The number of states for these analyses varies depending on age group, as some states reported mortality and custody information for one group (e.g., 49 years and under), but not another, depending on record-keeping processes. For states with information on those 49 years and under (*N* = 11; California, Colorado, Illinois, Iowa, Kansas, Maine, Maryland, Montana, New Mexico, North Carolina, and Ohio), the RR for 2020 compared to 2019 is 1.30 (CI: 1.09 to 1.56). For states with information for ages 50 to 64 (*N* = 8; California, Colorado, Illinois, Kansas, Maine, Montana, North Carolina, and Ohio), the RR for 2020 compared to 2019 is 1.71 (CI: 1.47 to 1.98). For states with information for ages 65 years and older (*N* = 8; California, Colorado, Illinois, Kansas, Maine, Montana, North Carolina, and Ohio), the RR for 2020 compared to 2019 is 1.58 (CI: 1.37 to 1.81). We also calculated a total mortality RR for this subgroup of states to assess how this group compares to the larger sample of 49 DOCs. This subgroup’s RR (RR = 1.75, CI: 1.60 to 1.91) is higher than the ratio for the larger sample, but negative binomial regression models produce similar coefficients across both samples (subsample: RR = 1.77, CI: 1.37 to 2.28; larger sample: RR = 1.77, CI: 1.62 to 1.93). The age-stratified RRs are lower overall compared to the total mortality RR; for example, the RR for people 65 years and older is 1.58, and the total RR (across all age groups) is 1.77. This is due to the changing age structure of the population in 2020 compared to 2019: Custody levels decreased in 2020 primarily among younger incarcerated people, leaving a higher proportion of older people (with higher mortality rates overall) in custody in 2020.

To further understand excess mortality at the youngest ages, we conducted additional analyses looking at variations in age groups with a smaller number of states with these data [*N* = 7; California, Colorado, Illinois, Maine, Maryland, Montana, and Ohio; note that Maryland is only available for the youngest age group (18 to 24 years), and Ohio is only available for the older groups (25 years and older)]. Using negative binomial regressions, the RR for 2020 compared to 2019 is 2.06 for ages 18 to 24 (CI: 0.78 to 5.48), 1.41 for ages 25 to 44 (CI: 1.08 to 1.84), and 1.56 for ages 45 to 49 (CI: 1.04 to 2.24). Note that for the youngest ages (18 to 24 years old), the number of deaths per year across the states in this sample is quite small (*N* = 10 in 2020, *N* = 9 in 2019, etc.), leading to large standard errors and CIs.

### Mortality by manner of death in 2020 compared to 2019

**Fig. 2.**
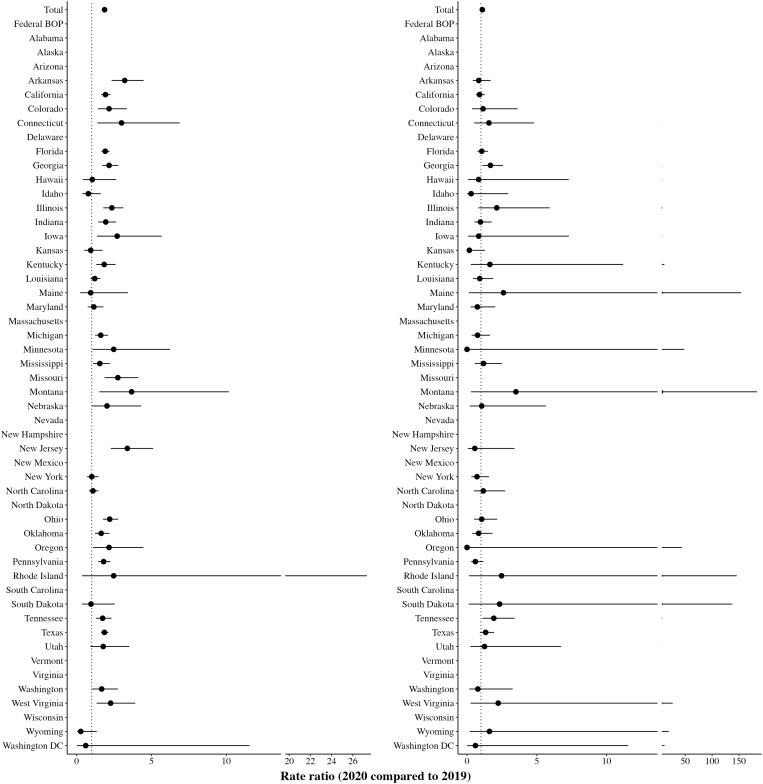
Mortality rate ratio, by natural (left) and unnatural (right) deaths.

Second, we assess how mortality rates by manner of death changed between 2020 and 2019. We first turn to estimates of natural deaths, due to COVID-19 and other medical causes. Across the 41 DOCs with these data (40 states and Washington, D.C.), the RR for 2020 compared to 2019 for deaths due to natural causes is 1.86 (CI: 1.77 to 1.96) (Fig. 2). These rates obscure substantial variation across states. Some states report lower mortality rates due to natural causes, even including COVID-19 deaths, in 2020 compared to 2019. For example, Hawaii, Idaho, Kansas, Maine, New York, North Carolina, South Dakota, Wyoming, and Washington, D.C. experienced mortality RRs of approximately 1 or lower for natural deaths in 2020 compared to 2019. Many of these states (Hawaii, Idaho, Kansas, New York, and North Carolina), however, have a high percentage of 2020 deaths (10% or more) marked as unknown, a point we analyze below. Other states (Colorado, Georgia, Illinois, Iowa, Minnesota, Ohio, Oregon, and West Virginia) experienced RRs at least two times higher, and, in some states (Arkansas, Connecticut, Montana, and New Jersey), at least three times higher, for deaths due to natural causes in 2020 compared to 2019. For deaths due to unnatural causes (e.g., suicide, accident, homicide, trauma, or overdose), the mortality RR for 2020 compared to 2019 across the 41 DOCs is 1.10 (CI: 0.97 to 1.24) (Fig. 2). The RR reflects substantial variation across states. States like Georgia (RR = 1.68, CI: 1.11 to 2.58) and Tennessee (RR = 1.92, CI: 1.11 to 3.41) report large increases in mortality rates for deaths due to unnatural causes. Other states (Maine, Minnesota, Montana, Oregon, Rhode Island, South Dakota, West Virginia, and Wyoming) have few (and often, no) unnatural deaths for any particular year; these low-rate states have large CIs.

Next, we calculate mortality by manner of death for 2020 compared to prior years (2013–2019) by estimating separate negative binomial regression models for deaths due to natural and unnatural causes (Table 1, models 2 and 3). Compared to the reference year of 2019, the 2020 RR for mortality due to natural causes is 1.80 (CI: 1.59 to 2.04, *P* < 0.001). Mortality rates due to natural causes stayed relatively constant from 2013 to 2019. For mortality due to unnatural causes, the RR is 1.08 (CI: 0.93 to 1.27, *P* = 0.308). Mortality rates due to unnatural causes increased over 2013–2016 and then plateaued in 2017–2019.

For these analyses distinguishing between natural (including COVID-19) and unnatural causes, many states have a large proportion of deaths labeled as unknown in 2020 (see table S4 for the proportion of unknown deaths from 2013–2020 by state). Excluding the 15 states with 10% or more unknown deaths in 2020, the RR for 2020 compared to 2019 for natural deaths and unnatural deaths, respectively, is 2.04 (CI: 1.92 to 2.16) and 1.22 (CI: 1.05 to 1.42). Results from negative binomial regressions, using this same subset of states with low proportions of unknown deaths, show that deaths from natural causes were nearly two times higher in 2020 (RR = 1.98, CI: 1.72 to 2.29, *P* < 0.001) compared to 2019. The RR for deaths from unnatural causes is 1.20 (CI: 1.00 to 1.45, *P* < 0.10).

### Mortality for deaths due to natural causes (without COVID-19) in 2020 compared to 2019

**Fig. 3.**
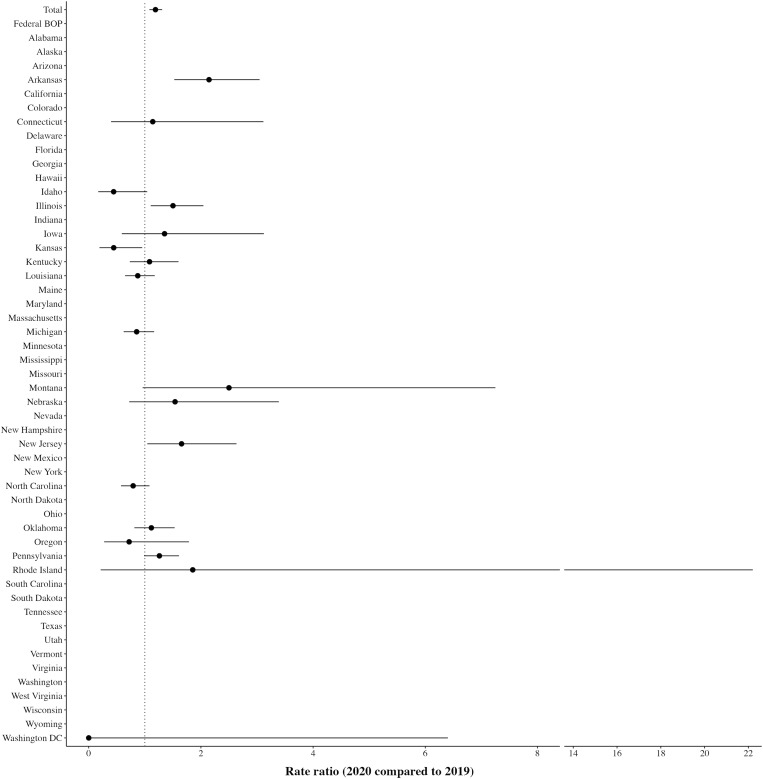
Mortality rate ratio for natural deaths excluding COVID-19 deaths.

Third, we estimate how mortality rates for deaths due to natural causes other than COVID-19 changed between 2020 and 2019. Across the 18 DOCs with these data (17 states and Washington, D.C.), the RR for 2020 compared to 2019 is 1.19 (CI: 1.08 to 1.31) for deaths due to natural causes without COVID-19 deaths (Fig. 3). A negative binomial regression model estimates an RR of 1.18 (CI: 0.96 to 1.44, *P* = 0.114). As with the previous analyses, there is substantial variation across states, with six states (Idaho, Kansas, Louisiana, Michigan, North Carolina, and Oregon) experiencing lower RRs for natural deaths without COVID-19 deaths in 2020 compared to 2019. However, these states also report at least 10% of deaths as unknown in 2020. A negative binomial regression model limited to the 12 states with smaller proportions of unknown deaths (Arkansas, Connecticut, Illinois, Iowa, Kentucky, Massachusetts, Montana, Nebraska, New Jersey, Oklahoma, Pennsylvania, and Washington, D.C.) estimates an RR of 1.44 (CI: 1.19 to 1.76, *P* < 0.001) for deaths due to natural causes (apart from COVID-19) in 2020 compared to 2019.

### Additional analyses

Custody populations declined in 2020 due to pandemic-related mitigation responses (with people released early in some states). To understand how this decline might matter, we replicated the regression analyses with lagged custody variables to understand excess mortality rates based on these alternative custody numbers. As expected, given the larger custody populations at the end of 2019 (as opposed to the end of 2020), the RRs for 2020 deaths (and end of 2019 custody) compared to 2019 deaths (and end of 2018 custody) are lower (RR = 1.54, CI: 1.40 to 1.70) than the ratios based on concurrent custody populations (RR = 1.77, CI: 1.62 to 1.93). This pattern is relatively consistent for mortality rates for natural deaths (RR = 1.51, CI: 1.30 to 1.75 for lagged custody and RR = 1.80, CI: 1.59 to 2.04 for concurrent custody), unnatural deaths (RR = 1.08, CI: 0.87 to 1.34 for lagged custody and RR = 1.08, CI: 0.93 to 1.27 for concurrent custody), and natural deaths without COVID-19 deaths (RR = 1.01, CI: 0.76 to 1.34 for lagged custody and RR = 1.18, CI: 0.96 to 1.44 for concurrent custody). These lagged analyses show the consequences of population shifts for RRs, but are likely underestimates compared to our primary analyses, given the decreasing custodial populations throughout 2020.

## DISCUSSION

We used unique data collected from public records requests, supplemented with information from state DOC websites and other public sources, to examine total mortality and mortality by manner of death among people incarcerated during 2020. This research advances knowledge about mortality in U.S. prisons in four ways. First, we provide a comprehensive accounting of mortality across nearly all 50 state prison systems, the Federal BOP, and Washington, D.C., producing the first comprehensive estimate of overall increasing mortality in prisons during the pandemic while simultaneously revealing important variation across states. Second, we conduct additional analyses by age group to understand age variation in total mortality in U.S. prisons. Third, we estimate all-cause mortality, mortality from natural causes, and mortality from unnatural causes, showing that even deaths due to unnatural causes increased in some states, providing a more accurate understanding of the total mortality toll among incarcerated people during the pandemic. Fourth, we distinguish among deaths from COVID-19 and other natural causes to show how deaths due to other natural causes also increased during 2020.

Across these contributions, we highlight several key findings. First, in regression models that account for clustering by state, we find that total mortality increased by 77% in 2020 relative to 2019. This mortality increase is strikingly higher than the 23% increase in mortality among the general population (*1*). Further, our age-stratified analyses suggest that excess mortality is not limited to the oldest age groups, yet simultaneously highlight the vulnerability of older people in prison. Analyses of the general population show that younger people (25 to 44 years old) experienced the highest percentage increase in mortality (27% in January–October 2020 compared to 2015–2019) (*24*). In our analyses of in-custody mortality, younger people experienced even higher percentage increases in mortality (41% increase relative to 2019), but the largest increases in mortality occurred among those ages 50 to 64 years (65% increase relative to 2019) and 65 years and older (58% increase relative to 2019).

Second, the total mortality increases in prison in 2020 obscure substantial variation between states, including mortality increases as much as two and three times higher in 2020 relative to 2019, in some states, like Illinois and New Jersey, respectively. Differences in characteristics of state prison systems, prison mitigation policies, and broader state contexts likely affected state variation in mortality (including deaths from COVID-19 and deaths due to other natural and unnatural causes). Analyses examining key state-level factors (imprisonment rate, a prison health care index) indicate that imprisonment rates (but not the state health care index) are positively associated with prison mortality rates, overall, but they are not additionally related to 2020 increases. Analyses examining general population COVID-19 positivity rates are positively associated with mortality. These findings suggest that prison staff remained important vectors of COVID-19 transmission, despite widespread prison visitor prohibitions and lockdowns in 2020 intended to prevent COVID-19 spread into prisons from surrounding communities (*10*). While additional factors certainly contributed to state-level variation in 2020 mortality rates (and further data collection, including month-level custody and death data, will facilitate theses analyses), documenting and analyzing deaths is a first critical step to understanding both the causes and the scope of the mortality consequences of COVID-19.

Third, COVID-19 mortality numbers alone severely understate the impact of the pandemic on people in prison. We find that non–COVID-19 mortality increased, including mortality due to unnatural causes in some states. Specifically, mortality rates due to unnatural causes increased substantially in Georgia (RR = 1.68, CI: 1.11 to 2.58) and Tennessee (RR = 1.92, CI: 1.11 to 3.41). Mortality due to natural causes apart from COVID-19 deaths also increased in some states. Across states with these data, the RR for natural deaths other than COVID-19 deaths is 1.18 (CI: 0.96 to 1.44, *P* = 0.114); however, excluding states with large proportions of deaths from unknown causes, the RR is 1.44 (CI: 1.19 to 1.76, *P* < 0.001). These steep increases suggest systemic failures that simultaneously increased risk of illness and limited access to medical care. During 2020, staff shortages, often resulting from pandemic-related illness, and constrained medical resources led to failures to meet the routine primary and specialty health care needs of incarcerated people (*20*, *21*). Pandemic-related mitigation policies and practices, including lockdowns and restricted movement, programming suspensions, visitor prohibitions, limited communication with loved ones, and solitary confinement in lieu of medical isolation, also increased stress, anxiety, and other mental health conditions (*21*, *22*). Moreover, the large number of causes of death reported as unknown suggests another systemic failure: inconsistencies and gaps in DOC reporting of mortality data. Troublingly, even in pre-pandemic years, some jurisdictions (e.g., California, Maryland, Missouri, New York, and Oregon) routinely reported a high prevalence of unknown causes of death.

### Limitations

These contributions should be interpreted with several limitations in mind. First, mortality data from DOCs are variable across states and subject to misreporting (particularly, underreporting) (*25*). In addition to the number of deaths reported as unknown, misreporting might also affect the accuracy of manner of death categorizations. Misreporting also likely affects classifications of whether deaths took place under state DOC jurisdictions; some jail systems, for example, systematically released terminally ill people before death to avoid counting them in death data (*26*). The extent this happened in state prison systems is unknown. Furthermore, a national accounting of deaths in these other carceral facilities (including jails, halfway houses, and immigration detention centers) is an important future research direction. Second, while this study focused on excess mortality in 2020, continuing to document and examine mortality in subsequent years is necessary to fully understand the mortality toll in prisons during the pandemic, as well as how more recent policy responses (e.g., vaccines) might have affected mortality. Third, despite the comprehensiveness of our state sample, we are missing all information for two states (Virginia and Wisconsin) and some data from other states. Fourth, examining differences due to race/ethnicity and gender, which rely on mortality and custody data consistently and accurately distinguishing these groups, is beyond the scope of this paper. Race/ethnicity, in particular, is often reported with extensive differences across states, requiring a focused analysis attendant to those variations. Because racial/ethnic disparities and gender differences are closely related to U.S. incarceration and COVID-19–related deaths (*27*), understanding pandemic-related total mortality in prisons by race/ethnicity and gender is critical for future work.

### Policy and practice implications

The findings have several implications for policy and practice. First, U.S. prisons are particularly vulnerable to COVID-19 and its collateral consequences for health. COVID-19 mitigation practices—systematic testing for incarcerated people and staff, vaccines, personal protective equipment, and reducing overcrowding to enable social distancing (*17*, *28*, *29*)—were especially limited in carceral facilities, where they were arguably most needed. According to the 1976 Supreme Court case of *Estelle* v. *Gamble*, “deliberate indifference” to the “serious medical needs” of people in prison is unconstitutional, violating the Eighth Amendment prohibition against cruel and unusual punishment; as recent lawsuits have argued, the health care failures inherent in the U.S. carceral response to COVID-19 are potentially constitutional violations (*23*, *30*). The disproportionate mortality rates documented here provide further evidence of these health care failures. Even despite the limited COVID-19 mitigation measures prisons did take, like visitor prohibitions, facilities remained vulnerable to general population COVID-19 rates, attesting to the porousness of prison walls, which officers and other staff pass through daily. Mitigation policies that focus on prison staff (including universal testing and vaccine mandates) appear particularly important (*11*). Better policies should be considered now to prevent future pandemics. Second, data transparency about deaths in carceral facilities is fundamentally lacking but desperately needed (*31*). Despite the Death In Custody Reporting Act, there is currently (2019 to present) no publicly available information about mortality in U.S. prisons. Not only is there an urgent need, especially during a global pandemic, for a full accounting from all 50 state prison systems (as well as the Federal BOP and Washington, D.C.), but reporting information about both manner of death and demographic data (race/ethnicity, gender, and age, at a minimum) is critical to understanding inequalities related to deaths in custody.

In a country with extremely high incarceration rates, fully understanding the mortality toll of the COVID-19 pandemic in the United States requires accounting for incarcerated people. Incarcerated individuals disproportionately experience health risks, endure economic disadvantage, and are more likely to be racial/ethnic minorities compared to the general population; consequently, the lack of transparency and data access about mortality in prisons is especially important to acknowledge and account for in studies of mortality and health inequalities.

## MATERIALS AND METHODS

We conducted a retrospective population-based (2013–2020) analysis of prison mortality in 48 DOCs (including 46 states, the Federal BOP, and Washington, D.C.) that provided information on annual counts of prison mortality and custody population throughout the time period. Additionally, 18 states (Alabama, California, Colorado, Delaware, Georgia, Kansas, Maine, Mississippi, Montana, Nevada, North Dakota, Oregon, Pennsylvania, Rhode Island, South Dakota, Texas, West Virginia, and Wyoming) provided monthly mortality and population information. Eleven states (California, Colorado, Illinois, Iowa, Kansas, Maine, Maryland, Montana, New Mexico, North Carolina, and Ohio) provided these data by age group, enabling age-stratified analyses. We obtained most data via public records requests, supplementing with publicly available data—including DOC statistical reports, public records requests made by other investigators, and third-party data sources (Texas Justice Initiative: https://texasjusticeinitiative.org; MuckRock: https://www.muckrock.com)—when necessary. This research is approved by the University of California, Irvine Institutional Review Board (HS#2021-6770), and data are archived in the Dataverse Project (*32*).

Our analyses of mortality rates rely on both death and population counts in prisons across state DOCs. We measure the number of deaths, overall and by manner of death, for each state per year. We measure mortality as total mortality, or the number of deaths from any cause, and manner of death as natural (deaths from medical causes, including COVID-19), unnatural (deaths due to suicide, accident, homicide, trauma, or overdose), and unknown. These counts exclude executions. We measure the deaths attributed to COVID-19 based on DOC-provided classifications. In the few states where deaths were reported with more detail (*N* = 5), deaths were categorized as attributable to COVID-19 if it was a contributory cause.

Preparing the original files required extensive data quality checking (to resolve inconsistencies in number of deaths reported by states across categories, ensure that the jurisdictional scope excluded deaths outside of prison, and identify duplicate deaths) and data management (e.g., creating standardized data cleaning notes and templates across states). We also spent considerable effort to harmonize variables across states, since departments provided data that varied in terms of available information, formats, and categorizations. In some cases, states provided record-level mortality with more detail than natural and unnatural, including notes on cause of death, which we coded into the natural and unnatural categories. For states that provided discrepant information about deaths, we deferred to the most recently collected data (for states that responded with multiple rounds of data); in one situation, we excluded a specific state-year (Hawaii-2015) from the analysis, because we could not resolve discrepancies, and the state did not respond to clarification requests.

In our primary models estimating excess mortality in 2020, we measure the custodial population for each state at year end (2013–2020). Five DOCs (Alaska, New Hampshire, North Dakota, South Carolina, and the Federal BOP) provided mid-year custody counts, and we used multiple imputation methods (*33*) to estimate year-end custodial populations for these states in regression analyses. Mortality rates per state and year are the number of deaths per 1000 people in the custodial population. Because custody populations changed more drastically in 2020 in some states due to pandemic-related intake and release policies, we also conduct supplemental analyses that measure mortality rates using a lagged year-end custody measure.

In additional analyses, we include two pre-pandemic state-level variables and one pandemic-related state-level variable to understand how these factors are associated with total mortality, both before and during the pandemic. First, we include a standardized measure of 2019 state imprisonment rates (a common indicator of state punitiveness in the criminal legal system) using publicly available data from BJS (*34*). Second, we create a prison health care index using publicly available data from two nationwide 2015 surveys conducted by The Pew Charitable Trusts and Vera Institute of Justice (*35*). For this index, we standardize and take the mean of the following: (i) the number of health professional full-time equivalents (FTEs) per 1000 incarcerated people, (ii) health care spending per capita, (iii) health care monitoring (monitoring with feedback, monitoring without feedback, no monitoring), and (iv) health care services model (direct, contracted, hybrid). These two pre-pandemic state-level variables facilitate a preliminary investigation of the hypotheses that punitive structural factors, along with inadequate health care, might have contributed to increases in total mortality. Third, we include general population state COVID-19 positivity rates (as reported by The COVID Tracking Project, https://covidtracking.com), hypothesizing that prison officers and staff might have been primary vectors of COVID-19 transmission to prisons, especially in 2020 when visitation and programming were largely suspended. For yearly models that include state positivity rates, we take the state average across 2020 months (March to December). For monthly models, we use state-month lagged measures of positivity rates.

### Statistical analysis

We estimate excess mortality for 2020 compared to 2019, presenting total mortality RRs overall and by state. We then calculate excess mortality for 2020 (compared to 2019 but also including 2013–2018 to examine broader trends) using a negative binomial regression analysis, in which deaths are predicted based on year fixed effects and exposure (custody) with clustered standard errors for states, with corresponding RRs and 95% CIs. We repeat this approach to examine excess mortality by manner of death and excess mortality for deaths due to natural causes other than COVID-19–related reasons.

In additional regression models, we examine pre-pandemic and pandemic-related state variables as control variables to estimate their associations with total mortality. We also conduct additional regression models that consider subsets of states with total mortality and custody information by age groups (to estimate age-stratified RRs) and that exclude states with larger numbers of unknown deaths (10% of more of their total deaths in 2020).
