# Supplementary material for: Excess mortality in U.S. prisons during the COVID-19 pandemic
Source: Sci Adv. 2023 Dec 1;9(48):eadj8104. doi: 10.1126/sciadv.adj8104 (PMC10691764; doi:10.1126/sciadv.adj8104)
Supplement: Supplementary file 1 — Tables S1 to S4 [file sciadv.adj8104_sm.pdf]

Science Advances
AAAS

Supplementary Materials for

Excess mortality in U.S. prisons during the COVID-19 pandemic

Naomi F. Sugie *et al.*

Corresponding author: Naomi F. Sugie, nsugie@uci.edu

Sci. Adv. **9**, eadj8104 (2023)
DOI: 10.1126/sciadv.adj8104

This PDF file includes:

Tables S1 to S4

Table S1. Data Sources and Years Observed.

	Mortality Data Source	Custody Data Source	Years Observed
Federal BOP	PRR	Public	2013-2020
Alabama	Public	Public	2013-2020
Alaska	PRR	Public	2013-2020
Arizona	PRR	Public	2013-2020
Arkansas	PRR	Public	2013-2020
California	Public	Public	2013-2020
Colorado	PRR	PRR	2013-2020
Connecticut	PRR	Public	2013-2020
Delaware	PRR	PRR	2015-2020
Florida	Public	Public	2013-2020
Georgia	PRR	Public	2013-2020
Hawaii	PRR	PRR	2013-2020
Idaho	PRR	PRR	2013-2020
Illinois	PRR	PRR	2013-2020
Indiana	PRR	Public	2013-2020
Iowa	PRR	PRR	2013-2020
Kansas	PRR	PRR	2013-2020
Kentucky	PRR	Public	2013-2020
Louisiana	Public/PRR	Public	2015-2020
Maine	PRR	PRR	2013-2020
Maryland	PRR	PRR	2014-2020
Massachusetts	PRR	Public	2013-2020
Michigan	PRR	Public	2013-2020
Minnesota	PRR	Public	2013-2020
Mississippi	PRR	Public	2013-2020
Missouri	PRR	Public	2013-2020
Montana	PRR	PRR	2013-2020
Nebraska	Public/PRR	PRR	2013-2020
Nevada	Public	Public	2013-2020
New Hampshire	PRR	Public	2013-2020
New Jersey	PRR	Public	2013-2020
New Mexico	PRR	PRR	2013-2020
New York	PRR	Public	2013-2020
North Carolina	PRR	Public	2013-2020
North Dakota	PRR	PRR	2013-2020
Ohio	PRR	Public	2015-2020
Oklahoma	PRR	Public	2015-2020
Oregon	PRR	PRR	2013-2020
Pennsylvania	PRR	Public	2013-2020
Rhode Island	PRR	Public	2013-2020
South Carolina	PRR	Public	2013-2020
South Dakota	PRR	Public	2013-2020
Tennessee	Public	Public	2013-2020
Texas	Public	Public	2013-2020
Utah	PRR	PRR	2013-2020
Vermont	PRR	N/A	2013-2020
Virginia	N/A	N/A	N/A
Washington	PRR	Public	2013-2020
Washington DC	PRR	Public	2013-2020
West Virginia	PRR	PRR	2013-2020
Wisconsin	N/A	N/A	N/A
Wyoming	PRR	PRR	2015-2020

Notes: Public records request is denoted as "PRR." Publicly available (Department of Corrections website, other publicly available repository) is denoted as "Public."

Table S2. Mortality Counts, Total and by Manner of Death, 2020.

	Total Deaths	Natural		Unnatural	Unknown
		Total	COVID-19		
Total	6,088	4,118	496	534	482
Federal BOP	582				
Alabama	198				
Alaska	14				
Arizona					
Arkansas	173	154	51	16	3
California	498	378		58	62
Colorado	68	61		7	0
Connecticut	29	21	13	8	0
Delaware	21				
Florida	601	515		65	21
Georgia	280	196		58	26
Hawaii	16	10		2	4
Idaho	34	14	6	1	19
Illinois	168	147	53	13	8
Indiana	143	121		22	0
Iowa	30	28	14	2	0
Kansas	38	21	11	1	16
Kentucky	92	88	36	4	0
Louisiana	151	116	32	16	19
Maine	7	5		2	0
Maryland	67	46		8	13
Massachusetts	41	36	10	3	2
Michigan	248	139	65	12	97
Minnesota	18	18		0	0
Mississippi	109	79		17	13
Missouri	130	89		0	41
Montana	26	22	7	3	1
Nebraska	30	25	6	4	1
Nevada	82				
New Hampshire	7				
New Jersey	95	90	46	2	3
New Mexico	47				
New York	100	59		12	29
North Carolina	140	96	26	13	31
North Dakota	7	5		2	0
Ohio	250	233		17	0
Oklahoma	139	123	39	13	3
Oregon	50	27	18	0	23
Pennsylvania	220	204	61	14	2
Rhode Island	8	4	1	2	2
South Carolina	121	99		16	6
South Dakota	11	9		2	0
Tennessee	163	123		38	2
Texas	697	609		66	22
Utah	36	24		4	8
Vermont	3				
Virginia					
Washington	47	43		4	0
Washington DC	2	1	1	1	0
West Virginia	46	38		3	5
Wisconsin					
Wyoming	5	2		3	0

Notes: States without information on manner of death did not report this information.

Table S3. Negative Binomial Regression Models Estimating Total Mortality, Yearly and Monthly Observations.

	Model 1: Yearly				Model 2: Monthly			
	Coef.	RR	95% CI	p-value	Coef.	RR	95% CI	p-value
2020 (ref=2019)	0.60	1.82	(1.66-1.99)	<.001	0.27	1.30	(1.16-1.46)	<.001
Imprisonment rate	0.23	1.26	(1.10-1.44)	<.001	-0.05	0.95	(0.82-1.10)	0.485
Prison health care index	-0.04	1.04	(0.81-1.14)	0.657	-0.12	0.89	(0.70-1.14)	0.350
General COVID-19 positivity rate	-0.02	0.98	(0.97-1.00)	<.10	0.03	1.03	(1.01-1.05)	<.01
2020*Imprisonment rate	-0.03	0.97	(0.88-1.08)	0.592	0.04	1.04	(0.90-1.20)	0.596
2020*Prison health care index	-0.09	0.92	(0.80-1.05)	0.199	-0.01	0.99	(0.94-1.05)	0.832
Constant	-5.62	0.00	(0.00-0.00)	<.001	-8.12	0.00	(0.00-0.00)	<.001
DOCs (N)			46				18	

Notes: For Model 1, the sample includes 46 Departments of Corrections (DOCs), which corresponds to 49 DOCs from Model 1 of Table 1 minus the Federal Bureau of Prisons, New Hampshire, and Washington D.C. (which are missing variables for imprisonment rate, health care index, and/or general COVID-19 positivity rate). For Model 2, General COVID-19 positivity rate refers to the average state positivity rate in the general population between March and December 2020 (Model 1) and lagged monthly state positivity rate (Model 2).

Table S4. Proportion of Unknown Deaths, by Year.

	2020	2019	2018	2017	2016	2015	2014	2013
Total	0.08	0.06	0.05	0.04	0.04	0.02	0.03	0.04
Federal BOP								
Alabama								
Alaska								
Arizona								
Arkansas	0.02	0.04	0.03	0.06	0.01	0.00	0.00	0.02
California*	0.12	0.16	0.12	0.09	0.04	0.01	0.00	0.00
Colorado	0.00	0.00	0.00	0.00	0.00	0.00	0.00	0.00
Connecticut	0.00	0.00	0.00	0.07	0.00	0.00	0.00	0.00
Delaware								
Florida	0.03	0.05	0.01	0.01	0.06	0.01	0.01	0.11
Georgia	0.09	0.08	0.06	0.11	0.25	0.32	0.29	0.25
Hawaii*	0.25	0.06	0.17	0.14	0.06		0.91	1.00
Idaho*	0.56	0.04	0.00	0.00	0.00	0.00	0.00	0.00
Illinois	0.05	0.05	0.01	0.01	0.02	0.00	0.00	0.00
Indiana	0.00	0.00	0.00	0.00	0.00	0.00	0.00	0.00
Iowa	0.00	0.00	0.00	0.00	0.00	0.00	0.00	0.00
Kansas*	0.42	0.06	0.00	0.00	0.00	0.00	0.00	0.00
Kentucky	0.00	0.02	0.00	0.03	0.07	0.00	0.07	0.05
Louisiana*	0.13	0.02	0.01	0.02	0.01	0.00		
Maine	0.00	0.00	0.13	0.00	0.00	0.00	0.10	0.00
Maryland*	0.19	0.20	0.21	0.24	0.10	0.07	0.04	0.03
Massachusetts	0.05							
Michigan*	0.39	0.01	0.01	0.00	0.02	0.05	0.09	0.02
Minnesota	0.00	0.00	0.14	0.00	0.00	0.00	0.00	0.00
Mississippi*	0.12	0.00	0.00	0.04	0.01	0.00	0.00	0.04
Missouri*	0.32	0.56	0.61	0.06	0.10	0.09	0.24	0.17
Montana	0.04	0.00	0.00	0.00	0.00	0.00	0.00	0.00
Nebraska	0.03	0.00	0.00	0.00	0.00	0.00	0.00	0.00
Nevada								
New Hampshire								
New Jersey	0.03	0.16	0.18	0.18	0.13	0.02	0.02	0.02
New Mexico								
New York*	0.29	0.17	0.15	0.26	0.15	0.06	0.06	0.08
North Carolina*	0.22	0.03	0.01	0.01	0.00	0.02	0.05	0.06
North Dakota	0.00	0.00	0.00	0.00	0.00	0.00	0.00	0.00
Ohio	0.00	0.00	0.00	0.01	0.00	0.01	0.00	0.00
Oklahoma	0.02	0.01	0.00	0.01	0.01	0.02	0.03	0.03
Oregon*	0.46	0.66	0.26	0.25	0.40	0.16	0.29	0.71
Pennsylvania	0.01	0.00	0.00	0.00	0.00	0.00	0.00	0.00
Rhode Island*	0.25	0.00	0.25	0.00	0.00	0.00	0.00	0.00
South Carolina	0.05	0.00	0.01	0.00	0.01	0.00	0.00	0.00
South Dakota	0.00	0.00	0.00	0.00	0.00	0.00	0.00	0.00
Tennessee	0.01	0.01	0.00	0.00	0.00	0.00	0.00	0.00
Texas	0.03	0.01	0.05	0.02	0.01	0.00	0.00	0.00
Utah*	0.22	0.00	0.06	0.00	0.00	0.00	0.00	0.00
Vermont								
Virginia								
Washington	0.00	0.00	0.00	0.00	0.00	0.00	0.00	0.00
Washington DC	0.00	0.00	0.00	0.00	0.00	0.00	0.00	0.00
West Virginia*	0.11	0.04	0.00	0.00	0.00	0.04	0.00	0.00
Wisconsin								
Wyoming	0.00	0.00	0.00	0.00	0.00	0.00	0.00	0.00

Notes: *States with at least .10 of deaths coded as "unknown" in 2020. States without information on unknown deaths did not report manner of death.
